# Isolated fungus ball in sphenoid sinus: tips and pitfalls of *T*_1_ hyperintense lesions

**DOI:** 10.1259/bjrcr.20170081

**Published:** 2018-01-10

**Authors:** Teresa Popolizio, Marco Perri, Rosario Francesco Balzano, Bilal Al-Badayneh, Roberto Izzo, Paolo Graziano, Giuseppe Guglielmi

**Affiliations:** 1 Department of Radiology, Ospedale Casa Sollievo della Sofferenza, San Giovanni Rotondo, Puglia, Italy; 2 Department of Radiology, Università degli Studi di Foggia Scuole di Specializzazione di Area Medica, Foggia, Puglia, Italy; 3 Department of Radiology, Ospedale Cardarelli, Naples, Italy; 4 Department of Hemathology and Medical Oncology, Unit of Pathology, Ospedale Casa Sollievo della Sofferenza, San Giovanni Rotondo, Puglia, Italy; 5 Department of Radiology, Università degli Studi di Foggia, Foggia, Puglia, Italy

## Abstract

Isolated sphenoid sinus fungus ball is a very rare condition. CT is the most used imaging investigation for diagnosis. In some cases, MRI may provide further information to evaluate the extracompartmental invasion. We report the case of an elderly female patient who presented with headache and a soft tissue mass filling the right sphenoid sinus on CT, misdiagnosed as simple sinusitis. After 1 year, with recrudescence of symptoms, brain MRI showed a hyperintense soft tissue mass on *T*_1_ weighted images within the right sphenoidal sinus; a new CT examination revealed calcifications within the mass. Surgical histological examination showed fungus ball. Fungal ball should be included in the differential diagnosis of *T*_1_ hyperintense lesions in the sphenoid sinus.

## Case presentation

A 62-year-old female patient was admitted in April 2016 in another hospital complaining of severe persistent deep throbbing headache in the right retro-orbital region, not responsive to common non-steroidalanti-inflammatory drugs. Neurological examination did not show any signs of focal deficit. Ophthalmic examination of fundus oculi revealed normally appearing retina with normal eye pressure. In that occasion, the patient underwent brain CT, which showed no significant alterations of brain tissue; instead, obliteration of right sphenoidal sinus was observed and it was referred to sinusitis. After 1 month of oral antibiotic and corticosteroid treatments, symptoms partially reversed, with only a latent headache persisting during the follow-up period; however, throbbing headache recrudescence and posterior nasal drip occurred later in March 2017, when the patient came to our attention.

## Investigations

**Figure 1.**
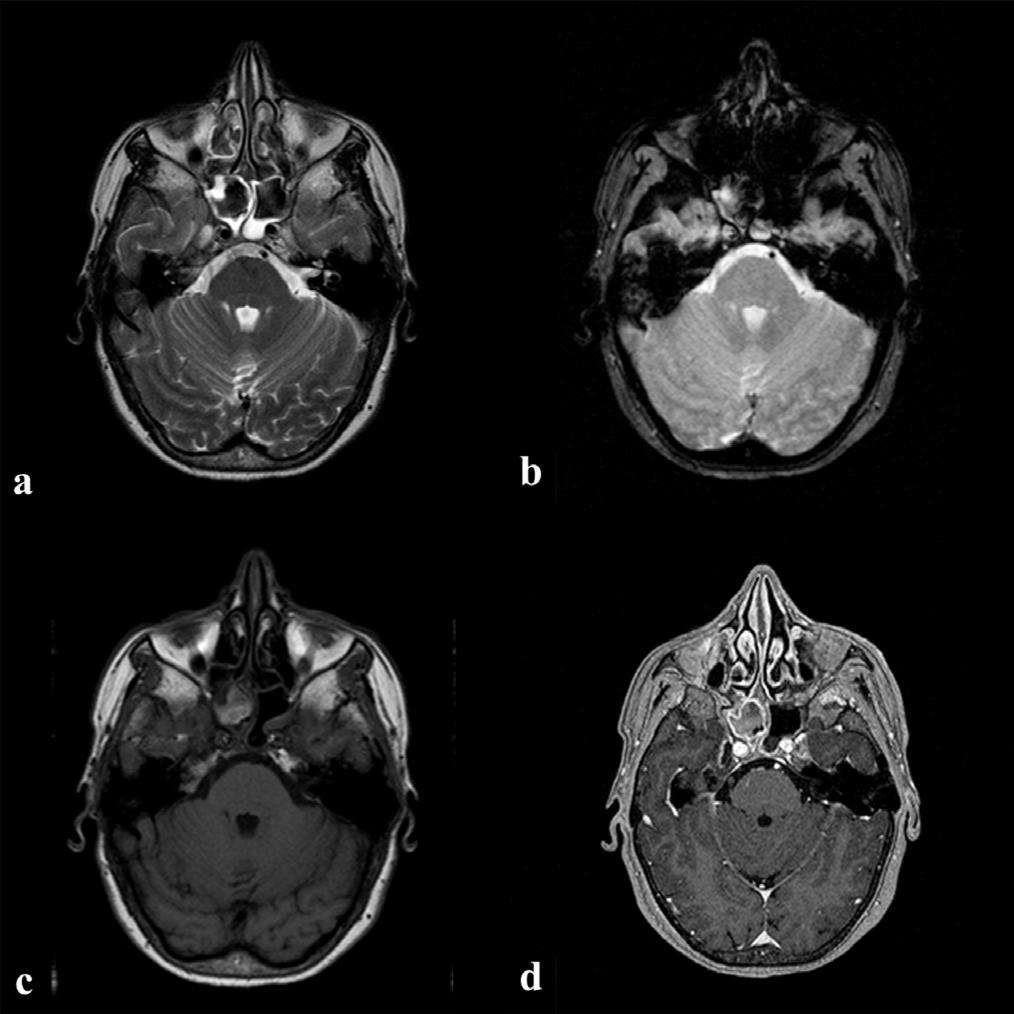
MRI examination: the right sphenoid sinus is filled with a soft tissue mass that appears hypointense on *T*_2_ weighted image (a) with some susceptibility blooming artefacts on *T*_2_* images (b) and is hyperintense on *T*_1_ weighted image; (c) on post-contrast MPRAGE T1 images (d) the mass did not enhance, whereas the inflammatory mucosa underwent Contrast Enhancement (CE).

**Figure 2.**
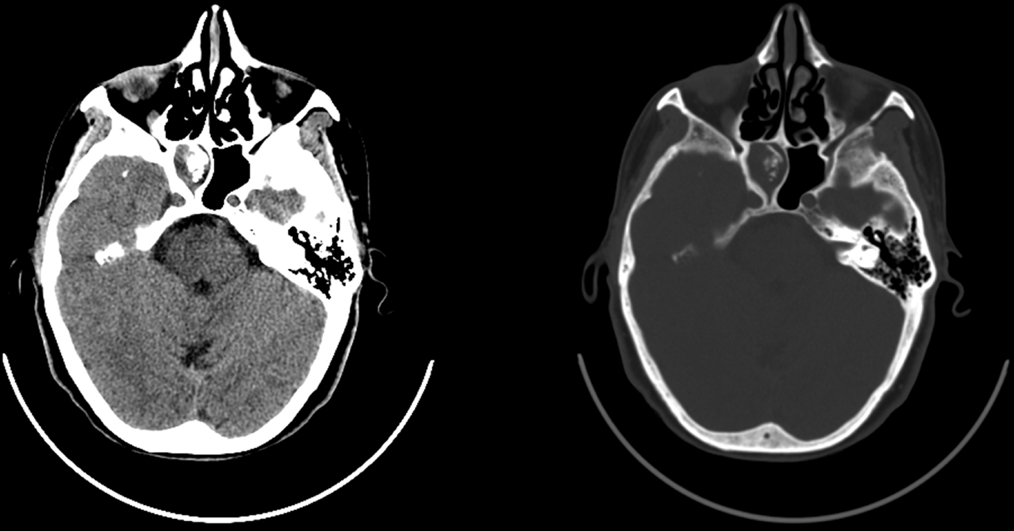
Axial CT image shows complete opacification of right sphenoid sinus with central calcified streaks. The bony wall appears bowed, sclerotic and thickened, without any signs of erosion or destruction.

We preferred to perform an MRI examination in order to better evaluate the brain and characterize the sphenoidal sinus pathology. MRI examinations were performed on a 1.5 MAGNETOM Aera scanner (Siemens Healthcare) with the following MRI parameters applied: SE *T*_1_, on transversal and sagittal planes, TR 450 ms, TE 8.90 ms, FA 90, thickness 5 mm, gap 6.50; BLADE T2, on transversal and coronal planes, TR 3800, TE 99, FA 141, thickness 5 mm, gap 6.5 mm; *T*_2_ FLAIR, TR 8500, TE 82, FA 150, thickness 5 mm, gap 6.5 mm; *T*_2_* GRE TR 830, TE 25, FA 20, thickness 5 mm, gap 6.5 mm, diffusion-weighted imaging, TR 7200, TE 81, FA 90, thickness 5 mm, gap 6.5 mm, *b* values 0 and 1000; post-contrast *T*_1_-MPRAGE TR 1900, TE 3.02, FA 15, thickness 1 mm, gap 0 mm. All the sequences were acquired using the same Matrix (320\320) and 1 NEX, except for diffusion-weighted imaging, in which 2 NEX was used. The MRI evaluation of the brain found only incidental chronic hypoxic lesions. The right sphenoid sinus showed irregular and hypertrophic T2 prolonged hyperintensity of the mucosa with central hypointensity (Figure 1a); this could be best appreciated as low signal on T2* GRE images (Figure 1b), and hyperintensity on *T*_1_ weighted images (Figure 1c). After contrast injection, there was enhancement of the right sphenoid sinus mucosa but not its contents (Figure 1d). The left sphenoid sinus showed minimal T2 hyperintense mucosal thickening due to reactive inflammatory changes but no evidence of left sphenoidal sinus disease or extracompartmental extension. To rule out the involvement of the surrounding neurovascular structures and bone infiltration, i.v. administration of paramagnetic contrast medium was injected. In order to evaluate bone wall status and exclude erosions, we performed a new CT scan (Toshiba Aquilion 64 CT Scanner) that revealed total obliteration of the right sphenoid sinus with some contextual streaks calcifications (corresponding to *T*_2_* blooming artefacts on MRI) and sclerotic bone wall without continuity into the cortical bone(Figure 2). The other paranasal sinuses showed minimal mucosal thickening with tiny calcifications but no bony thickening, sclerosis or erosion. Laboratory tests revealed no significant abnormalities concerning immunity level or any other significant result. Based on these radiological findings we hypothesized the diagnosis of chronic non-invasive fungal infection (FB) of the right sphenoidal sinus.

### Differential diagnosis

As far as we know, FB with a central *T*_1_ weighted hyperintensity has not been well described in the literature. Owing to this peculiar appearance, the differential diagnosis covered the following: mucocele, which with its protein content commonly demonstrates elevated signal intensity on *T*_1_ weighted images but also appears hyperintense on *T*_2_ weighted images and does not show enhancement; inverted papilloma, but it usually involves the nasal cavity (mostly the middle meatus) and secondarily affects sinuses; sinonasal mucosal melanoma, whose *T*_1_ hyperintensity is due to haemoglobin derivatives from previous bleeding, but it shows contrast enhancement and generally arises from the nasal cavities; mature nasal osteoma, for the presence of central fatty marrow that is hyperintense on *T*_1_ weighted images; FB.^1, 2^ Our case was previously diagnosed as simple sphenoid sinusitis only on the basis of CT examination; on the contrary, our MRI examination revealed in the right sphenoid sinus a hyperintense hypertrophic mucosa on *T*_2_ weighted images, and a central hypointense signal alteration; for this reason, we excluded inverted papilloma and mucocele. The *T*_1_ weighted sequence documented a slight hyperintense signal intensity soft tissue mass within the sinus: this finding may be explained by the presence of tiny calcification in the tissue matrix.^2^ The mass did not show CE on fat suppressed *T*_1_ weighted images and, for this reason, we excluded melanoma; on the other hand, the sinus mucosa was enhancing. On *T*_2_* images blooming artefacts were noticed in the central part of the lesion and they were compatible with the gross calcifications detected on the CT scans; consequently, mature osteoma was not taken into account. No further significant pathological alterations were found in the other paranasal sinuses. The final diagnosis was that of isolated right sphenoid FB. Treatment is surgical, using two endoscopic approaches: transethmoidal and transnasal. The latter is considered easier and faster with less complications. A good preoperative assessment is mandatory because if a dehiscence of lateral sinus wall is noticed on radiological examinations, fungal hyphae may spread to the surrounding neurovascular structures during the surgical intervention, causing life-threatening complications. In order to minimize this possibility, medical treatment should be started about 4 weeks before surgery.^3, 4^ Endoscopic surgical removal of the fungal mass with re-establishing sinus drainage and irrigation to clear fungal debris is the goal of management. Recurrence is rare; however, it can be seen as late as 2 years.^4^ After surgery, our patient experienced a complete remission of symptoms.

### Treatment and outcome

**Figure 3.**
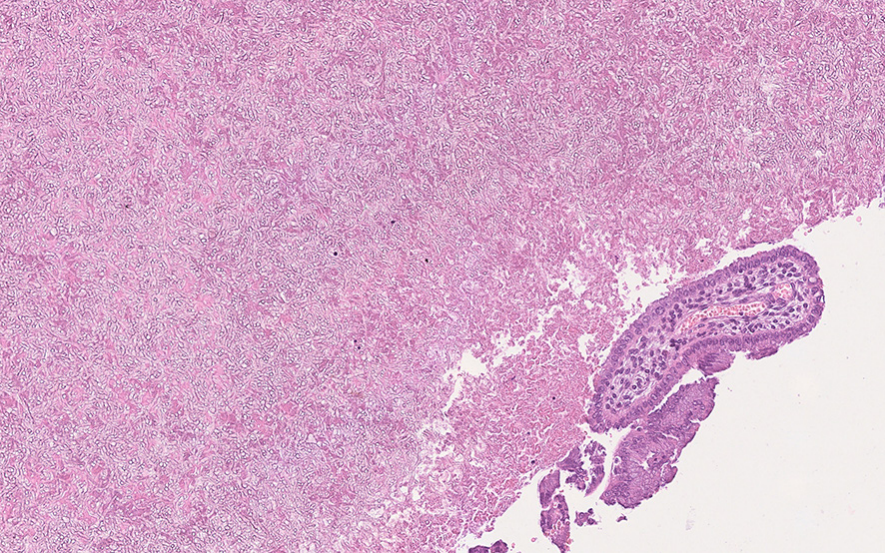
Histopathological image reveals fungal hyphae consistent with Aspergillus fumigatus (H & E 22x).

**Figure 4.**
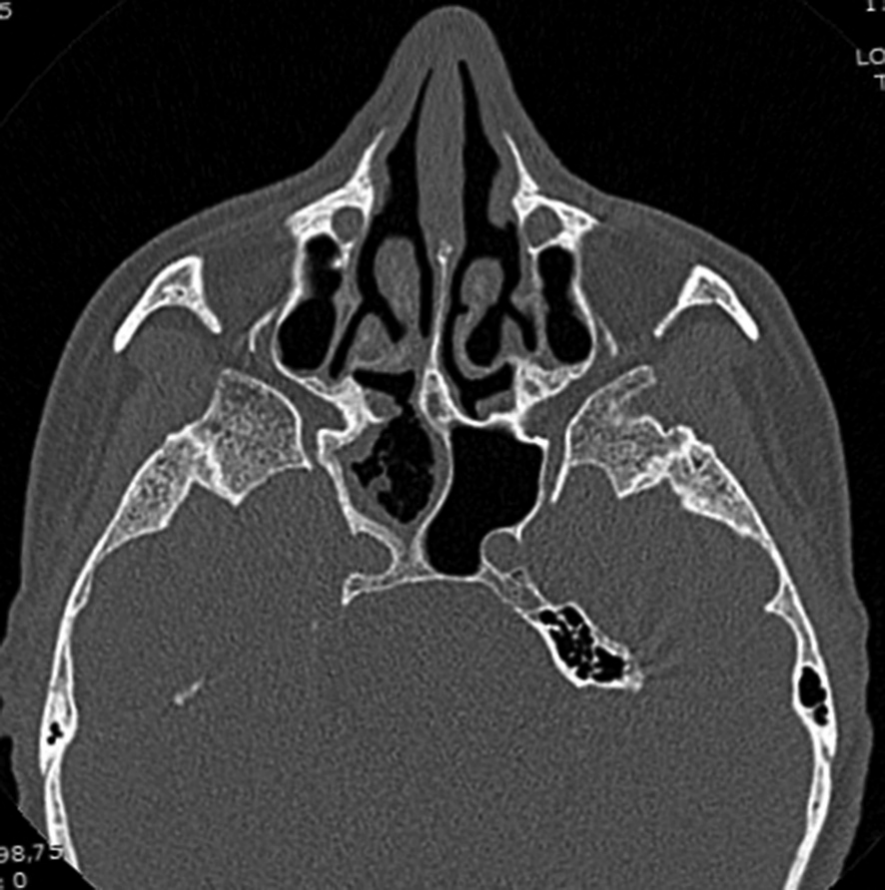
Axial postoperative CT image shows disappearance of right sphenoid sinus mass with only residual mucosal thickening, while sclerosis, thickening and bowing of right sphenoid sinus bony walls were still present with no evidence of dehiscence.

The patient underwent transnasal approach endoscopic surgery with mucosal curettage and establishing of a drainage opening. Histopathological examination confirmed the diagnosis of right sphenoidal sinus FB caused by Aspergillus fumigatus (Figure 3). Postoperatively, the patient clinically improved with gradual relief of her previous headache; a postoperative CT scan showed disappearance of the previously described right sphenoidal mass with no evidence of residual pathological tissue (Figure 4).

## Discussion

Fungal rhinosinusitis (FRS) is one of the most common pathologies that affects paranasal spaces, and radiological imaging plays an important role in its identification.^5, 6^ FRS is distinguished into two categories: invasive, which usually affects immunosuppressed patients and is characterized by the infiltration of the mucosa and spreading to the surrounding structures; non-invasive FRS, typically affecting immunocompetent patients, with no evidence of tissue invasion.^5, 7^ Paranasal sinus FB was first described in the 18th century and defined as extramucosal accumulation of dense conglomeration of fungal hyphae in a solitary sinus cavity.^8^ FB usually affects elderly immunocompetent patients (mean age of 64 years), with a prevalence in the female sex.^7, 9,10^ Several causes have been implied in its pathogenesis. The main pathological agents that sustain the infection are Aspergillus spp., but others pathogens have been also detected (such as Mucor, Alternaria and Bipolaris).^5^ Many misleading terms are found describing this condition such as mycetoma, aspergilloma and aspergillosis. Mycetoma is defined as chronic local invasive infection of the subcutaneous tissue that extends to adjacent structures, mostly seen in the hands and feet; with regard to aspergillosis and aspergilloma, Aspergillus species is responsible for many cases of invasive and allergic fungal sinusitis. In addition, in some cases of FB the culture is negative for Aspergillus and other fungal species, so these terms should be avoided and FB should be used instead.^10^ FB is found in about 3.7% of operated chronic inflammatory conditions affecting all paranasal sinuses. It is not a contagious disease and more commonly affects maxillary sinus— 83 to 88%—(the second most involved site is sphenoid sinus, 15%).^5, 11^ The maxillary sinus colonization may be due to odontogenic origin, as a consequence of dental procedures, but it would not explain the sphenoid localization and the occurrence of FB in patient with no previous dental interventions;^10, 12,13^ another theory, the so-called “aerogenic” theory, postulates that the paranasal sinuses may be infested by spores inhalation. It is possible that other predisposing factors are implied in FB development, such as host immunological state, concomitant pathologies (diabetes), oestrogens and anatomical variants.^5, 10,12^ Isolated sphenoidal sinus FB is a relatively rare entity that is more frequent in elderly female patients and may present with vague symptoms.^6^ Until 1992, only 21 cases of isolated sphenoid sinus FB were reported and this might have been due to its vague presentation and lack of advanced imaging tools. Nowadays, the incidence is increasing.^3^ Owing to its clinical presentation, the diagnosis of FB is usually delayed in time; in fact, it has been noticed that only 29% of cases of paranasal FB are diagnosed within 1 year of onset of symptoms and even much later in cases of isolated sphenoid sinus FB;^13^ presence of refractory chronic sinusitis symptoms should then raise the suspicion for fungal infection.^8, 14^ Blood tests are usually not contributory because the patients generally have no history of diabetes, immunosuppressive conditions or atopy.^10^ Clinically isolated sphenoid FB mostly manifests with headache, which radiates to the frontal, retro-orbital or occipital region in the case of sphenoid FB;^3, 11^ other reported symptoms are those of chronic nasal inflammation (posterior nasal drip, rhinorrhoea, nasal obstruction, hypo-/cacosmia) that does not improve with conventional antibiotic therapies.^3, 5,12^ Radiologically, CT and MRI examinations are the most suitable imaging modalities for its diagnosis as well as for pre- and postoperative evaluation, while conventional X-rays give non-specific findings.^15^ On CT scan FB typically appears as a hyperdense mass (due to matted fungal hyphae) usually limited to a single sinus, with linear or punctuate central calcifications. Thickened and sclerotic bony sinus walls with bowing deformity are also common without any signs of invasiveness. The thickened inflamed mucosa may enhance after iodine contrast administration.^1, 8^ On MRI, FB usually presents intermediate/low signal intensity on *T*_1_ weighted images and frank low signal intensity on *T*_2_ weighted images; blooming artefacts, due to the presence of calcifications, could be appreciated on *T*_2_* images. While the inflammatory mucosa appears hyperintense on *T*_2_ weighted images and may enhance as well, this latter aspect is best appreciated using *T*_1_ weighted images with fat suppression.^1, 13,15^

## Conclusions

FB should be suspected in refractory sinus diseases or in long-standing vague symptoms in older individuals. CT scan is the modality of choice for diagnosis, and MRI should be done if bone involvement is suspected; however, there are no pathognomonic imaging features and this is why histopathological confirmation should be done postoperatively.^6^ Generally, functional endoscopic sinus surgery is the modality of choice for management with no further medical treatment required. In our opinion, on the basis of previous considerations, this pathology may be underdiagnosed on routine evaluation and sometimes wrongly interpreted as simple sinusitis, with possible life-threatening complications. In our case, the diagnosis was also validated by the singular sign of unusual T1 hyperintensity signal of the fungal mass.

## Learning PoInts

1. FB is a very common disease usually correlated to Aspergillus thickness infections, which mostly involve the maxillary sinus; sphenoid sinus colonization is an uncommon event. Symptoms may be very vague and the diagnosis can be delayed, leading to complications such as venous thrombosis and visual disturbances.
2. On CT, FB appears as a hyperdense mass filling a sinus cavity with thickening and sclerosis of sinus walls; on MRI, it usually appears as a non-enhancing hypointense mass on *T*_1_ weighted image, but the presence of tiny calcifications within the lesion may confer hyperintense signal.
3. Surgical endoscopy is the treatment of choice, with expected complete remission of symptoms .

## Consent

Written informed consent for the case to be published (including images, case history and data) was obtained from the patient(s) for publication of this case report, including accompanying images.
